# Resolution of Sleep Apnea After Radiofrequency Ablation of Goiter

**DOI:** 10.1155/crie/6446712

**Published:** 2025-05-05

**Authors:** Kamran A. Ali, Daniel X. Ma, Lindsay M. McCullough, James J. Herdegen, Sean M. Wrenn

**Affiliations:** ^1^Department of Surgery, Rush University Medical Center, Chicago, Illinois, USA; ^2^Rush Medical College, Rush University Medical Center, Chicago, Illinois, USA; ^3^Department of Internal Medicine, Rush University Medical Center, Chicago, Illinois, USA

**Keywords:** goiter, radiofrequency ablation, sleep apnea, thyroid

## Abstract

**Background:** Obstructive sleep apnea (OSA) and nontoxic multinodular goiter are conditions that often coexist. Treatments of both conditions have evolved over time, but continuous positive airway pressure (CPAP), oral appliances, or surgical therapy are often needed. Radiofrequency ablation (RFA) of the soft palate and base of tongue has been applied as a newer alternative therapy for OSA. RFA is also an increasingly used approach for thyroid nodules and goiter, but previously had no known connection to OSA.

**Case Presentation**: A 59-year-old female with a known history of multinodular goiter and moderate OSA was referred to our endocrine surgery clinic. The goiter was found to have mediastinal extension, documented longitudinal growth of the dominant nodule, cosmetic deformity of the neck, and tracheal deviation. The patient underwent thyroid RFA as nonoperative treatment for her goiter. Within a month of her procedure, she also self-reported a subjective reduction in apneic events and later underwent a formal home sleep study demonstrating an apnea-hypopnea index (AHI) change from 15.8/h at diagnosis to 2.9/h currently, signifying resolution of her OSA. Her treated nodule had 92% volume reduction on 18-month follow-up visit.

**Conclusion**: To our knowledge, this is the first reported case of OSA cured in a patient undergoing RFA for goiter. Goiter-associated sleep apnea remains inadequately described in the literature and warrants further investigations on prevalence and management. Thyroidectomy continues to be the definitive treatment for goiter, with some studies suggesting secondary efficacy for OSA. RFA is now established as a first-line option for symptomatic thyroid nodules, but previously had no described benefit to OSA symptoms. This report illustrates that RFA of thyroid nodules could be offered to patients as both an effective nonsurgical option for goiter as well as a potential cure for their OSA to free them from nightly CPAP usage.

## 1. Introduction

Obstructive sleep apnea (OSA) is a chronic medical condition characterized by repetitive upper airway collapse during sleep. It is highly prevalent, with studies suggesting more than 20% of women and 40% of men exhibit sleep-disordered breathing symptoms [1]. Upper airway collapsibility, anatomical factors (including craniofacial structure, neck circumference, and obesity), arousal threshold, and ventilatory instability are the four major causes of OSA. Untreated OSA diminishes quality of life and increases the risk of hypertension, stroke, heart failure, diabetes, car accidents, and depression [1]. First-line treatment for OSA patients continues to be continuous positive airway pressure (CPAP), with other options ranging from oral appliances to surgical upper airway modification. While CPAP is the preferred therapy, approximately one-third of patients exhibit poor tolerance along with minor adverse effects, including mucosal dryness and skin irritation [2]. In the literature, there have been studies investigating the efficacy of radiofrequency ablation (RFA) of the soft palate and base of the tongue as an alternative therapy for mild to moderate cases of OSA [3–6]. Targeting these anatomic structures assists in relieving upper airway obstruction and preventing collapse of the pharyngeal muscles. RFA can conveniently be performed in the clinic without the need for general anesthesia and consequently has been applied as a second-line treatment for mild to moderate OSA when patients cannot adhere to or tolerate CPAP [6].

Beyond airway anatomy and obesity, various thyroid pathologies, including hypothyroidism and multinodular goiter, have also been associated with OSA [7]. The nodules can provoke OSA through mass effect on the airway, disrupting the function of the upper respiratory musculature. Hypothyroidism specifically also leads to mucopolysaccharide deposition in upper airway tissues, leading to inhibited function. Thyroidectomy has been shown in smaller cohort studies to have a secondary benefit in treating OSA as demonstrated through sleep studies and questionnaires, but nevertheless remains an invasive procedure [8]. Thyroid RFA has also been used as a nonoperative and minimally invasive approach to goiter that, thus far, has no published therapeutic relationship with OSA. Here we describe a patient with OSA who presented with multinodular goiter and underwent thyroid RFA with subsequent resolution of her sleep apnea. This suggests that RFA could potentially be offered as a minimally invasive option for goiter-associated sleep apnea to reduce nodule volume with fewer risks and quicker recovery times versus traditional surgery.

## 2. Case Presentation

### 2.1. Patient Information

**Figure 1:**
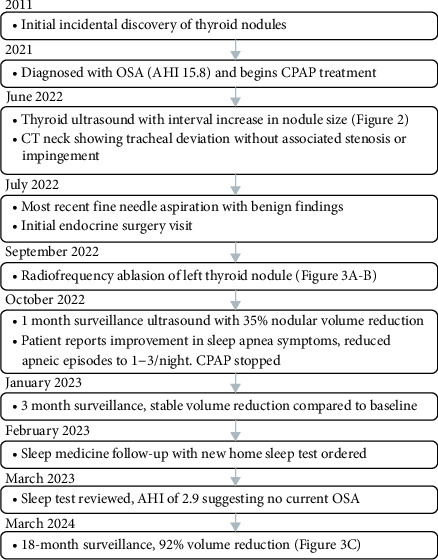
Timeline of patient encounters and pertinent findings.

**Figure 2:**
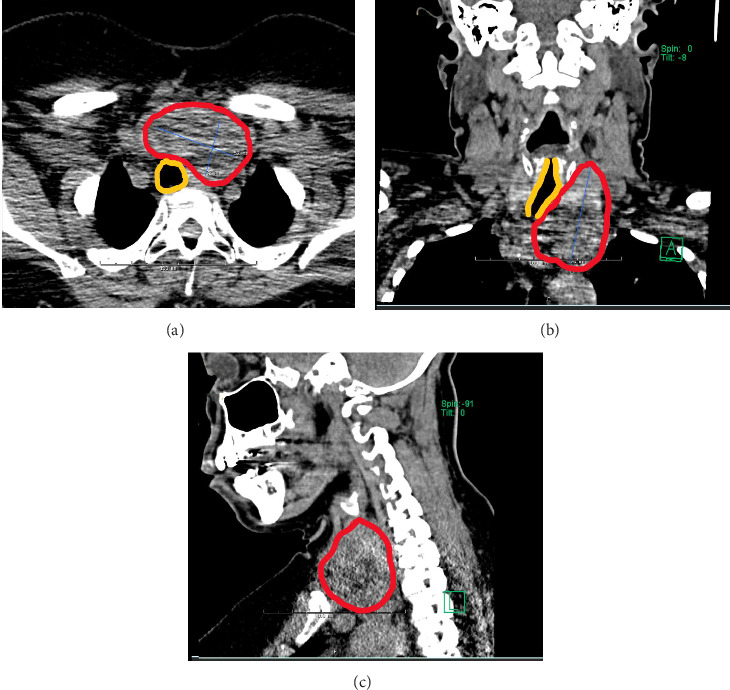
Computed tomography (CT) without IV contrast study showing thyroid nodule with substernal involvement (red outline). Trachea (yellow outline) deviated without compression due to the size and location of the nodule. (A) Axial view. (B) Coronal view. (C) Sagittal view.

A 59-year-old female presented to the endocrine surgery clinic in 2022 for evaluation of nontoxic multinodular goiter (Figure 1). She had been incidentally found to have thyroid nodules in 2011, which had been followed over time with serial imaging. More recent computed tomography (CT) and ultrasound (US) studies in 2021–2022 had demonstrated interval increases in nodule size and cervicothoracic tracheal deviation without tracheal stenosis or impingement (Figures 2A and 2B). Multiple fine needle aspiration biopsies had been performed with only benign (Bethesda class II) findings. She had not required any thyroid medications and denied any significant hypo or hyperthyroid related clinical symptoms. She also denied any voice changes, dysphagia, odynophagia, regurgitation, or reflux. There was a family history of thyroid disease, including her sister undergoing a thyroidectomy and her great-grandmother having goiter. Past medical history included obesity (BMI 31.6), hypertension well controlled on medications, anxiety, and OSA on CPAP. Her OSA was diagnosed by home sleep test in 2021 with an apnea-hypopnea index (AHI) of 15.8/h which classified it as moderate severity. For the 8:02 (h:min) of total testing time, an O_2_ nadir of 85% was reported with 0.4% of testing time spent below 90% room air. She had no prior history of neck surgeries or interventions. She had no history of smoking or tobacco use. She was clinically and biochemically euthyroid with a serum TSH of 0.700.

The patient expressed interest in a procedure to address her goiter given the evidence of nodular growth. However, she had an adverse experience with general anesthesia during a hysterectomy in 2011 and strongly wished to avoid surgery. Therefore, ambulatory RFA of the nodules was instead offered.

### 2.2. Clinical Findings

Clinical examination revealed an enlarged, mobile, nontender thyroid with palpable nodules. The goiter was easily visible in any neck position. Voice was normal with no hoarseness or weakness. There was no palpable or radiologic cervical lymphadenopathy. There was no Pemberton's sign. No oversized tonsils or tongue indentations were noted on physical exam. TSH was within normal limits.

### 2.3. Diagnostic Assessment

The patient had been routinely following up with her providers to assess the size of the nodules through US. Multiple fine needle aspirations had also been conducted and repeatedly yielded benign findings.

The most recent preprocedural CT neck study without contrast was performed in June 2022 (Figure 2) and was notable for a diffusely enlarged thyroid with a dominant left lobe. There were multiple nodules visualized and substernal extension into the superior mediastinum. The cervicothoracic trachea was deviated to the right without stenosis.

Thyroid US in June 2022 measured a smaller left thyroid nodule in the upper pole, around 1.8 cm × 1.2 cm × 2.2 cm. A larger nodule measured in the isthmus and lower pole of the left thyroid around, 4.8 cm × 3.0 cm × 6.9 cm. These represented increases in size compared to a previous US performed in March 2021.

A second fine needle aspiration in July 2022 was again negative for malignant cells, with cytology more consistent with benign follicular nodules.

After initially presenting to our clinic in late July 2022, the patient underwent a repeat thyroid US for volumetric analysis as part of our procedural planning. Here we measured the larger nodule as 5.22 cm × 3.16 cm × 7.06 cm with a volume of 58 mL. Volumes and percentage changes are calculated using the American Thyroid Association's online nodule volume calculator (https://www.thyroid.org/professionals/calculators/thyroid-with-nodules/). We also performed a preoperative indirect flexible laryngoscopy in August 2022 and noted normal bilateral vocal fold mobility and normal arytenoid cartilage. There was true vocal fold mobility with phonation. There was an extensive discussion about thyroid surgery, which was the initially recommended course, however, the patient was highly motivated to avoid surgery due to her desire to avoid permanent scarring and potential lifelong medication, along with previous adverse experiences with general anesthesia. The patient was approved for RFA of the large left thyroid and isthmus nodule.

### 2.4. Therapeutic Intervention

**Figure 3:**
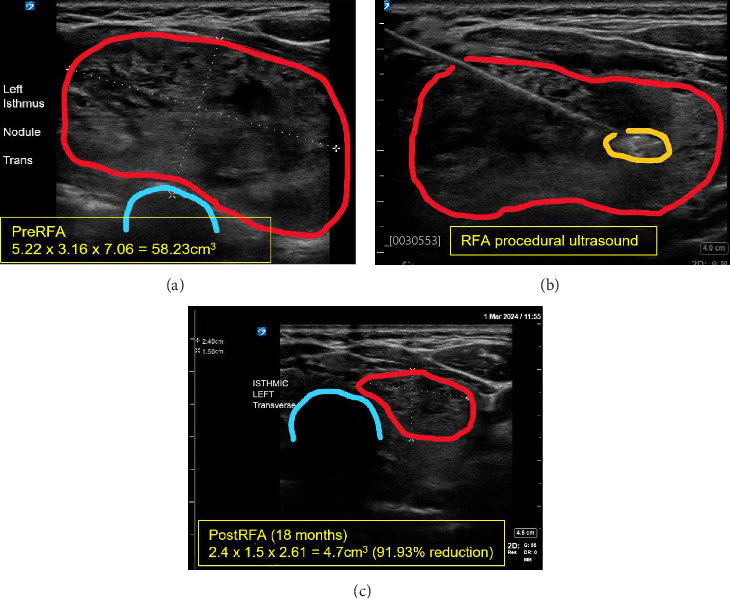
Ultrasound (US) imaging of left isthmus thyroid nodule. (A) Preradiofrequency ablation (RFA) transverse image of nodule (red outline) with trachea (blue outline) nearby. (B) RFA procedural US with thyroid nodule (red) and actively ablating tissue at needle point (yellow outline). (C) 18-month post RFA US showing reduction in nodule (red) near trachea (blue). Pre and PostRFA measurements included (units in cm).

The patient consented to RFA of the left thyroid and isthmus nodule and underwent the procedure in September 2022 (Figures 3A and 3B). 1 h prior to the ablation, she was given a one-time 1 mg oral dose of alprazolam for prophylactic anxiolysis as per standard protocol. Skin in the target area was pretreated with topical numbing spray. The patient was positioned supine in the clinic, and her neck was gently extended and then prepped and draped with chlorhexidine and sterile drapes. An US probe was used to locate the target nodule on the left side. 15 mL of 1% lidocaine with epinephrine was injected on the planned needle track and around the left thyroid capsule, which was the only hydrodissection required for the procedure. A Taewoong 10 mm active tip, 7 cm length, 18-gauge disposable radiofrequency probe was selected for this case. Grounding pads were placed, and the cooling circuit was established. Utilizing a trans-isthmic approach and the “moving shot” technique [9], we sequentially ablated the dominant nodule. The procedure was performed by author SMW, who has completed fellowship training in endocrine surgery, independent training in RFA, and is an experienced (>150 RFA cases) interventional thyroidologist.

Satisfactory ablation was determined by the measure of impedance rise, as well as radiological characteristics (e.g., bubbling). This nodule was done in a sweeping fashion from inferior/medial to lateral/superior, taking care to avoid the “danger” triangle near the nerve insertion. The needle path was visualized during ablation with special attention paid to the active needle tip. The maximum wattage used during the procedure was 60 watts with 22:46 (min:s) of total ablation time, which also closely matched the total operation time. Total energy was not recorded due to limitations in the software technology at the time.

During the procedure's critical portion, the patient was asked to vocalize to assure that no acute voice change or hoarseness was observed. The patient overall tolerated the procedure very well. Once the left side appeared satisfactorily ablated, we carefully removed the electrode. It appeared that with neck extension and optimal positioning, even the substernal portion of the nodule was able to be ablated successfully with complete visualization of the nodule. The “danger triangle” region was not specifically ablated due to high risk of potential recurrent laryngeal nerve injury, as per best practice guidelines. Pressure was held for several minutes after the removal of the electrode. Following the procedure, the patient was discharged home after 1 h observation with a plan to follow up in 1 month's time. The patient was advised to apply ice intermittently to the affected area, and to take ibuprofen and acetaminophen as needed for soreness and pain.

### 2.5. Follow-Up and Outcomes

The patient continued to be followed up through March 2024 in our clinic. She felt well at her 1-month follow-up in October 2022, with her neck feeling smaller and no vocal or skin changes noted. She denied any worsening of her sleep apnea symptoms in the initial recovery period from 1 week to 1 month postprocedure. US demonstrated a volume reduction of 34.5% compared to her baseline scans done before ablation (Table 1). At this visit, the patient also pointed out that her apnea symptoms appeared to have improved drastically. She reported 1–3 apneic episodes triggering per night versus roughly 19 around the time of her initial sleep study. Her sleep hygiene improved with occasional nights with 1–2 awakenings, but otherwise no difficulty initiating or maintaining sleep. Subjectively, she also felt much more energized during the day. Consequently, she elected to self-discontinue CPAP usage.

She returned in January 2023 with the US showing a relatively stable volume decrease. Table 1 reports a slight increase in volume and reduction in volume versus baseline compared to the October 2022 timepoint, which can be explained by variability in US measurements between different operators. At this visit, she felt her sleep apnea was resolved and so we placed a sleep study referral to definitively assess this.

**Figure 4:**
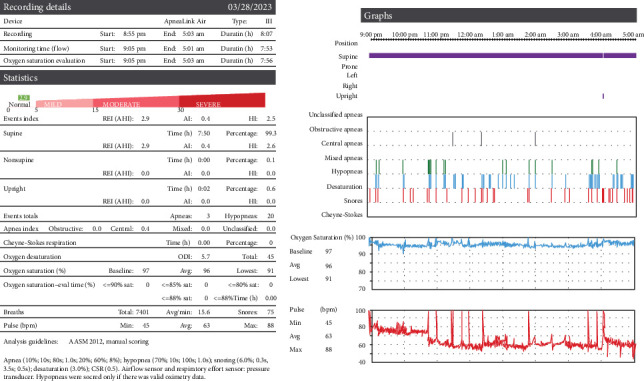
March 2023 sleep medicine study demonstrating AHI of 2.9/h.

Sleep medicine saw her in February 2023 and ordered a new home sleep test to compare with her original sleep study. The results were reviewed in March 2023 (Figure 4), and her AHI was 2.9/h, down from 15.8/h originally. The new O_2_ nadir was 91%, which was an improvement from 85% at the initial test. According to the index, she had successfully gone from moderate sleep apnea (AHI 15–30) to normal with no apnea (AHI < 5).

The patient was last seen in our clinic in March 2024 and continues to feel well and pleased with the RFA results. She no longer notices any contour deformity of her neck. US at this visit showed a nodule volume reduction of 92% from her baseline scan (Figure 3C).

## 3. Discussion

This is the first publication describing the use of thyroid RFA in successfully treating goiter-associated OSA. RFA is well-tolerated procedure that is safe and significantly less expensive than thyroidectomy [10]. Multiple studies have demonstrated that RFA is a viable and effective minimally invasive alternative to surgery for managing benign thyroid nodules that cause local symptoms or cosmetic issues [10–12]. However, surgery has been found to be more effective than a single RFA session for autonomously functioning nodules [13]. Additionally, RFA does not allow for final pathology, necessitating yearly post-op follow-up for at least 5 years [13].

When assessing goiter-associated OSA, there must still be consideration of all possible etiologies beyond thyroid causes of sleep apnea. Sleep apnea risk factors include overweight/obesity (BMI 26+), type 2 diabetes, pulmonary hypertension, congestive heart failure, atrial fibrillation, hypertension refractory to medications, and prior stroke [14, 15]. Increased neck circumference greater than 16 in. in women or 17 in. in men has also been associated with increased risk for OSA [16]. Finally, hypothyroidism is well known to also worsen OSA through pharyngeal narrowing via deposition of mucopolysaccharides and protein [17]. Other types of thyroid pathology are not as well studied for their relationships with sleep apnea.

In particular, there continues to be relatively little information available on the relationship between goiter and OSA. Goiters can have substernal involvement and, as they grow, can cause compressive symptoms including orthopnea, dysphagia, and voice changes (e.g., hoarseness) [18]. Multinodular goiter, large follicular adenomas, and Hashimoto's thyroiditis are some well-known benign etiologies of thyroid nodules. With sufficient size, these nodules can facilitate collapse of the upper airway during sleep. One pertinent study by Haddad et al. [19] reported 17 of their 24 goiter patients having polysomnographic evidence of OSA. This study found goiter-associated tracheal compression to be a statistically significant physical exam parameter that could distinguish between OSA and nonOSA patients. A systematic review on the relationship between goiters and surrounding anatomic structures noted tracheal compression in 9%–58% of cervical goiters and 35%–73% of substernal goiters [20]. We infer that this data suggests substernal involvement increases the risk for OSA. Overall, the increased prevalence of OSA in goiter patients provides further justification to intervene and ablate or excise the nodules.

While thyroidectomy remains the “gold standard” treatment option [21], RFA has become one of the most popular alternatives for addressing benign thyroid nodules. It is currently also considered a first-line treatment for benign thyroid nodules [22]. There are various thyroid RFA clinical guidelines that consider symptoms or cosmetic issues, hormone functional status, and biopsy findings to determine sufficient indication for RFA versus thyroidectomy [23]. However, at this time, OSA is currently not identified as a specific indication for thyroid surgery or RFA.

Our approach to RFA of the thyroid included interval quantitative (volumetric) assessments of the targeted (left thyroid-isthmus) nodule. For this patient, we were able to demonstrate a robust response to the RFA with a 92% reduction in nodule size at her most recent clinic visit. This process also allowed us to routinely meet with her to examine the neck and check in on her symptoms. We were able to verify that the goiter had shrunk considerably, both by our assessments as well as her own perspective. By bringing the sleep apnea to our attention early, we were able to regularly follow up on her symptoms and also coordinate with sleep medicine to organize an official sleep study. Her AHI change from 15.8/h to 2.9/h demonstrated resolution of her sleep apnea. Her comorbidities were relatively simple and well controlled. Her goiter was the most significant risk factor. In this case, she appeared to have a substernal goiter with tracheal deviation but no evidence of compression. The only other identifiable risk factor was obesity. Her BMI was noted to be 28.7 at her initial sleep study from before we first encountered her. It then increased to 31.6 on the first visit in our clinic and continued to fluctuate between 30 and 33 while we followed her course in subsequent visits. While the STOP BANG criteria for OSA is BMI > 35, weights falling into the overweight BMI category have also been found to be associated with OSA, with higher weights further worsening apneic severity [24, 25]. However, in this case, her worsening BMI did not appear to contribute to her OSA.

This case report had some limitations. The inherent nature of thyroid US measurements makes them operator dependent. The targeted nodule was primarily suprasternal with some substernal involvement, making it US (and RFA) accessible; we may have opted for surgery or another approach if that was not the case. In addition, the only reference AHI we had was her original diagnostic one from 2021, so it is possible that the severity of her sleep apnea had already been improving before the procedure. It is also important to note that the pathophysiology of OSA is multifaceted; anatomical causes are important but may not be the sole contributor to the condition.

From our literature review, it is clear there needs to be more studies investigating the relationship between goiter and OSA. The specific anatomic relationship of the thyroid and trachea presents a unique etiology of sleep apnea separate from other causes. Furthermore, there is clearly a difference in presentation and management of hypothyroid-associated goiters versus euthyroid nodular conditions. Conditions like multinodular goiter and follicular adenomas would not be amenable to medical management and may ultimately require procedural interventions. For these patients, RFA could offer a minimally invasive option with excellent nodule reduction.

This case presents the first known usage of thyroid RFA in treating both multinodular goiter and sleep apnea. Further organized studies should be conducted to investigate the relationship between goiter and OSA with specific assessments of nodule volumes versus OSA severity. These could then be followed by additional studies comparing the efficacy of RFA versus thyroidectomy in managing these patients. The authors suggest screening patients who present with goiter for a history of OSA or OSA-related symptoms. In select patients, RFA could be a potential minimally invasive alternative for goiter-associated OSA, allowing patients to avoid surgery while also possibly freeing themselves of nightly CPAP usage after confirmatory follow-up testing.

## Figures and Tables

**Table 1:** | Changes in thyroid nodule volume over 18 months.

Ultrasound date	Length (cm)	Width (cm)	Depth (cm)	Volume (cm^3^)	Change vs baseline (%)
Baseline Sept 2022	5.22	3.16	7.06	58.23	—
Oct 2022	5.91	2.73	4.73	38.16	−34.47
Jan 2023	5.27	3.01	5.2	41.24	−29.17
Mar 2024	2.4	1.5	2.61	4.70	−91.93

## Data Availability

Data sharing is not applicable.
